# The role of digital breast tomosynthesis in the breast assessment clinic: a review

**DOI:** 10.1002/jmrs.230

**Published:** 2017-04-04

**Authors:** Suneeta Mall, Sarah Lewis, Patrick Brennan, Jennie Noakes, Claudia Mello‐Thoms

**Affiliations:** ^1^ Faculty of Health Sciences University of Sydney Lidcombe New South Wales Australia; ^2^ Northern Sydney & Central Coast BreastScreen Royal North Shore Hospital St. Leonards New South Wales Australia

**Keywords:** Assessment, biopsy, breast cancer, DBT, diagnosis, digital breast tomosynthesis

## Abstract

Mammography has long been considered as the primary technique in breast cancer detection and assessment. Despite low specificity, mammography has been preferred over other contemporary techniques such as magnetic resonance imaging (MRI), computed tomography (CT) and ultrasonography (US) due to superior sensitivity and significant health economic benefits. The development of a new technique, a limited angle cone beam pseudo‐three‐dimensional tomosynthesis, digital breast tomosynthesis (DBT), has gained momentum. Several preliminary studies and ongoing trials are showing evidence of the benefits of DBT in improving lesion visibility, accuracy of cancer detection and observer performance. This raises the possibility of adoption of DBT in the breast cancer assessment clinic, wherein confirming or dismissing the presence of malignancy (at the potential site identified during screening) is of utmost importance. Identification of suspected malignancy in terms of lesion characteristics and location is also essential in assessment. In this literature review, we evaluate the role of DBT for use in breast cancer assessment and its future in biopsy.

## Introduction

Mammography has been widely adopted as the primary screening tool in breast cancer detection and assessment. The biggest challenge in mammography continues to be the ability to adequately represent the complex three‐dimensional (3D) breast architecture and its subtle anatomical changes in a two‐dimensional view. Mammography delineates a representation of the breast without a quantifiable measure of depth. This not only limits the visibility of lesions by increasing the obscurity from overlapping tissue (the ‘overlap effect’),^1^ but may also cause distortion in lesion characteristics due to firm compression.^2^ Inherent low X‐ray attenuation differences between cancerous and non‐cancerous tissues in dense breasts may further contribute to inferior contrast resolution in mammography. These limitations result in reduced lesion visibility and may adversely affect the decisions made by readers. Due to these shortfalls, the range of sensitivity of mammography is 70–90% and specificity is 60–80%.^3^

**Figure 1:**
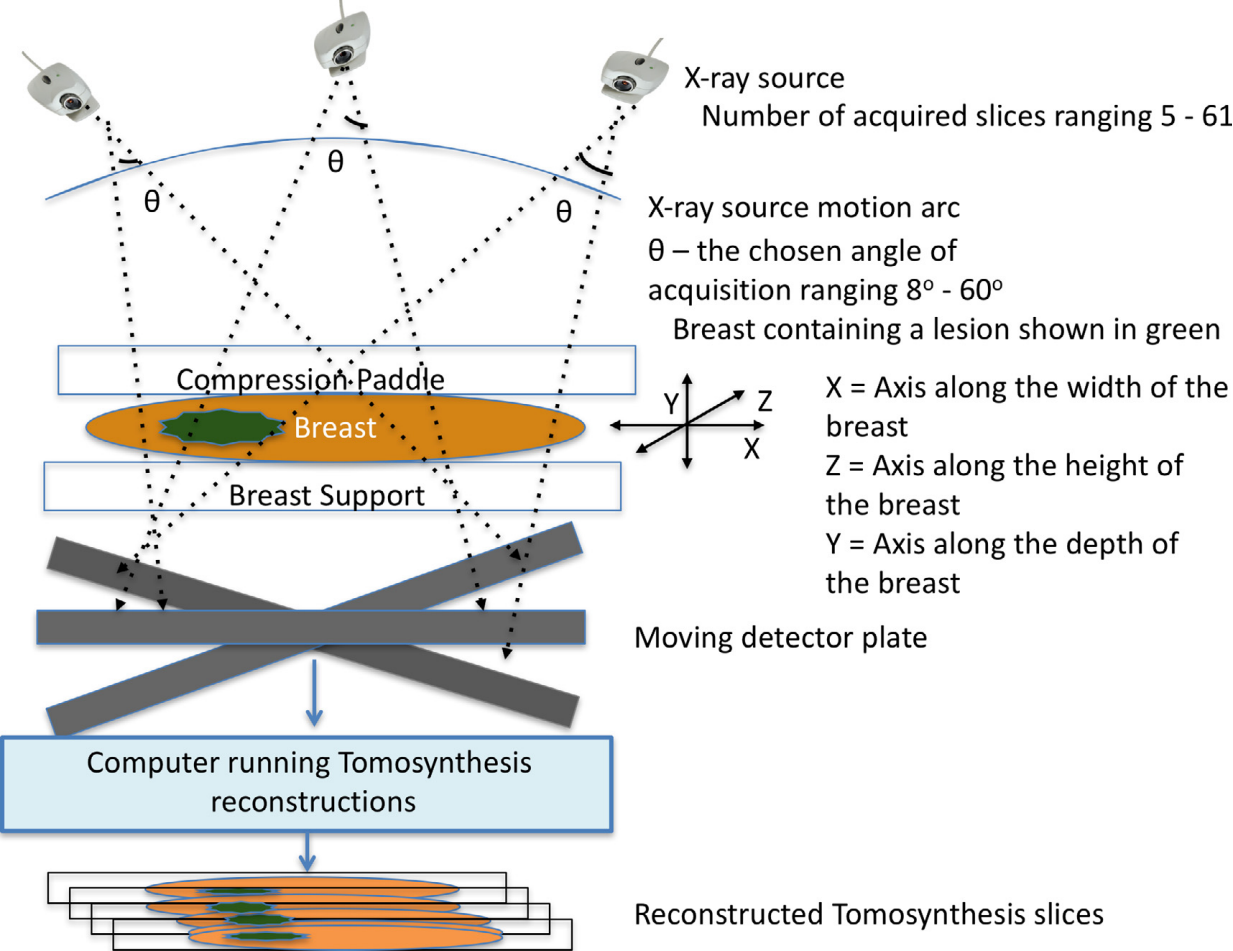
Schematic of standard digital breast tomosynthesis (DBT) system where an X‐ray source moves in an arc and steps and shoots an X‐ray beam that falls on the breast compressed between the compression paddle and the support plate. The digital breast image obtained for each acquisition of the moving X‐ray source is then processed via a software program to obtain 3D‐like DBT images.

Digital breast tomosynthesis (DBT) (Fig. 1), a pseudo‐three‐dimensional X‐ray imaging technique, has recently been integrated into clinical use. The results of several pilot studies^4^, ^5^, ^6^, ^7^, ^8^, ^9^, ^10^, ^11^, ^12^, ^13^, ^14^, ^15^, ^16^, ^17^, ^18^, ^19^, ^20^, ^21^, ^22^, ^23^ and trials^15^, ^16^, ^24^, ^25^ have suggested that DBT not only has the potential to substantially eliminate the tissue ‘overlap effect’^1^ but also can potentially reduce recall rates at screening (~17%),^4^, ^14^, ^15^, ^21^ improve lesion visibility,^4^, ^6^, ^7^, ^8^, ^10^, ^19^, ^21^ increase cancer detection (~51% (total),^15^ 40% (invasive cancers)^26^, ^27^), increase diagnostic accuracy^13^, ^14^ and improve patient comfort.^1^, ^28^

**Figure 2:**
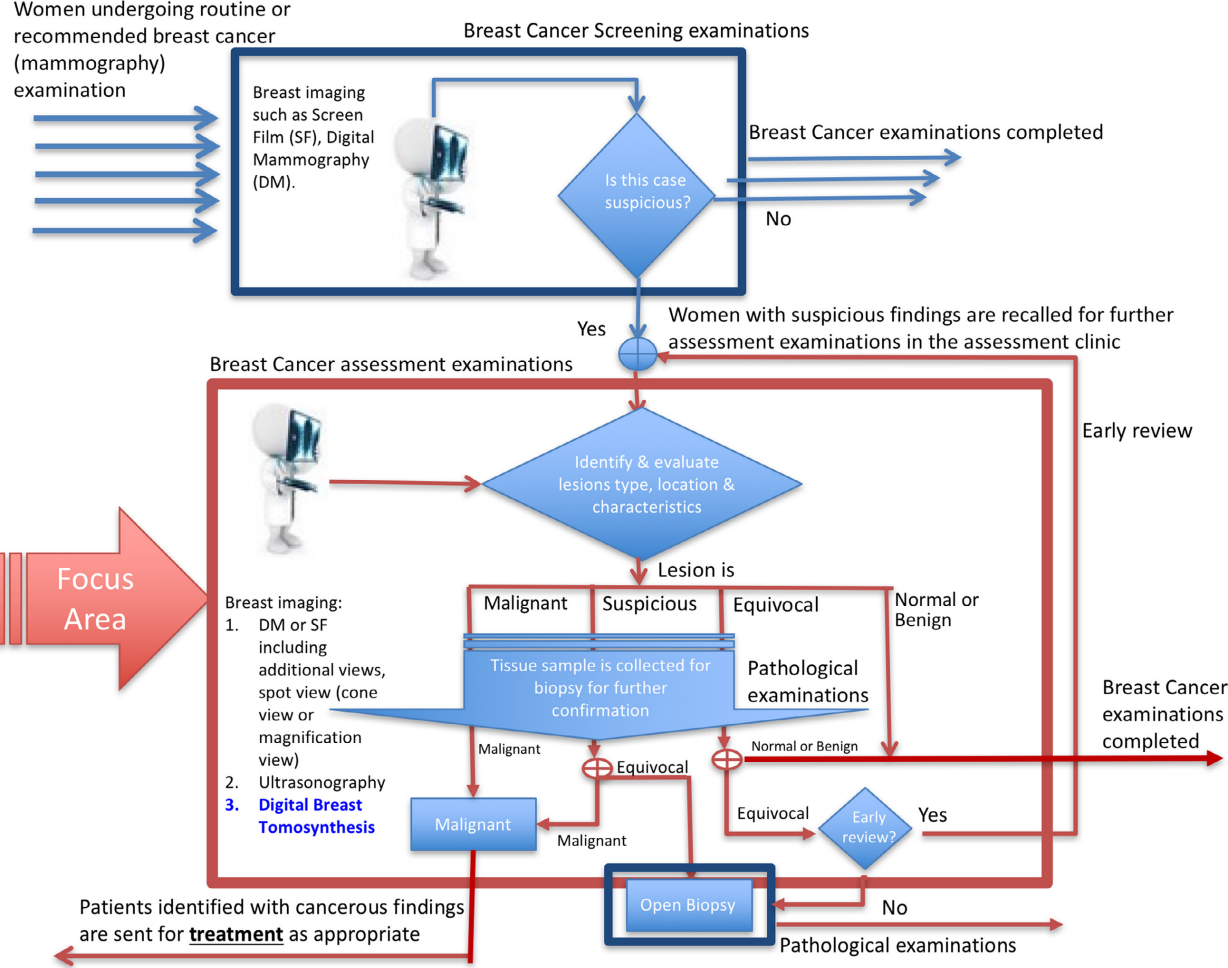
Workflow of screening and assessment scenarios, describing the steps involved in diagnosing breast cancer. The screening process is the first step in breast cancer detection, followed by assessment if suspicious findings are identified during screening.

Most of the above studies focused primarily on the use of DBT for breast cancer *screening*. The primary objective of screening is cancer detection, that is, identifying the presence of possible malignancy (Fig. 2). This is essential to the purpose of screening, which includes preventive health care and early detection of carcinoma. In breast cancer *assessment*, the focus is shifted towards confirming or dismissing the presence of malignancy at the site of the potential abnormality detected at screening (Fig. 2). The role of assessment in cases of suspected malignancy involves lesion localisation, identification of lesion characteristics (including margins and extent) and cancer type (with information obtained by imaging‐guided percutaneous biopsy) (Fig. 2). Additionally, in equivocal cases where no discrete lesion amenable to biopsy is identified, assessment may include recommendations for an early review on a case‐by‐case basis, enabling a second look at the abnormality. The effect of use of DBT in such scenarios is the focus of this literature review.

Initially, women undergo breast cancer assessment after being recalled for further examination due to presence of suspicious findings during screening. Hence, the prevalence rate (PR) of cancer in assessment (PR ≥ 10%; 16%^29^ to 49%^30^) is much higher than that of screening (PR~1%; 0.8%^15^). The anticipated probability of the presence of malignancy, if known, impacts perception and search strategies^31^ of the readers thereby impacting the reporting of the borderline lesions.^32^ Accurately identifying normal and benign lesions to reassure cancer‐free women is also crucial in assessment. Very few studies^12^, ^17^, ^27^, ^29^, ^30^, ^33^, ^34^, ^35^, ^36^, ^37^, ^38^ have focused on evaluating the efficacy of DBT in assessment. Due to scarcity of literature, the advantages of use of DBT in assessment are not well understood; however, the results of DBT in screening appear promising and raise the possibility of the successful use of DBT in assessment clinics.

## Methods

We reviewed articles from 1997 till 2016 that have specifically evaluated (1) use of DBT in assessment clinic, (2) use of DBT in biopsy, (3) compared DBT with workup‐view and/or spot‐view mammography (SVM) and (4) focused on lesions’ characteristics. Our search criteria consisted of keywords ‘breast cancer assessment’, ‘breast diagnosis’, ‘breast lesion characterization’, ‘breast biopsy’, ‘masses’, ‘calcification’ that were used together ‘DBT’ (and ‘tomosynthesis’ in a separate attempt). The searches were performed on three databases: (1) Web of Science, (2) Cross‐Search (University's search database) and (3) Google Scholar.

## Results: Use of DBT in Assessment Clinic

### Efficacy: sensitivity, specificity

Several assessment‐based studies^4^, ^5^, ^6^, ^7^, ^8^, ^9^, ^10^, ^11^, ^12^, ^13^, ^14^, ^15^, ^16^, ^17^, ^18^, ^19^, ^20^, ^21^, ^22^, ^23^, ^27^, ^37^ have been conducted to compare the performance of DBT with digital mammography (DM)^5^, ^6^, ^7^, ^8^, ^9^, ^10^, ^11^, ^12^, ^13^, ^14^, ^15^, ^16^, ^18^, ^19^, ^20^, ^21^, ^22^, ^23^ and SVM,^17^ either in a standalone mode (DBT alone)^4^, ^5^, ^6^, ^7^, ^8^, ^9^, ^10^, ^11^, ^12^, ^13^, ^14^, ^15^, ^16^, ^17^ or in combined mode (DBT as an adjunct to DM or SVM).^5^, ^18^, ^19^, ^20^, ^21^, ^22^, ^23^, ^30^

An early study has noted that 75% of the radiologists deemed DBT in combined mode with DM to be comparable or superior to mammography workup views in an assessment clinic.^12^ A consensus for the diagnosis of atypical ductal hyperplasia and intraductal papilloma lesions was also noted, with 100% of readers reporting DBT in combined mode with DM to be comparable or superior to a mammography workup scenario.^12^ DBT also reportedly simplifies the workup^2^ and is comparable to spot‐view mammogram.^2^, ^17^ Waldherr et al.^20^ observed an increased sensitivity and specificity of 91.9% and 87.5%, respectively, by using DBT in combined mode with DM for symptomatic women while evaluating lesion visibility and detection rate of these two techniques. The reported sensitivity and specificity of DBT (88.7%, 93.8%) in stand‐alone mode, however, was predominantly higher as compared to DM.^20^ Also, one‐view DBT was found to be superior for dense breasts when compared with two‐view DM.^38^ DBT has also been linked with increased radiologists’ confidence in the assessment clinic.^37^

A study focusing primarily on distinct assessment of breast cancers, reported statistically significant improvement in radiologists’ diagnostic performance with DBT (combined mode with DM) as compared to DM alone.^30^ This study reported an increase in sensitivity (both overall and lesion location specific), specificity, area under receiver operating curve characteristics (AUC) and jackknife free‐response receiver operating characteristic figure of merit.^30^ Another study^39^ reported similar results.

A specificity value of 60%^21^ for DM in screening suggests that for every three women correctly diagnosed as being cancer free, two women will be falsely suspected as potentially having cancer and will undergo unnecessary further diagnostic assessment that may include percutaneous tissue biopsy (core or fine needle) in addition to the workup views. The diagnostic workup generally requires at least one additional form of imaging such as SVM, US or MRI. It has been noted that the false positive diagnosis in screening leads to unnecessary examinations at a rate of 21% for biopsy and 50% for US.^29^ This not only increases the patient anxiety and reduces the cost effectiveness of screening but may also increase the risk of additional radiation exposure (due to workup views). Preliminary results from the Victoria (Australia) trial suggest that use of DBT in assessment could provide a statistically significant reduction in the rate of unnecessary biopsy (from 16.7% without DBT to 11.8% with DBT) and a reduction in the need to use ultrasound imaging (from 58.0% to 50.1%).^29^ Similarly, initial results from a UK trial^36^ suggests that DBT increases the accuracy of assessment when used as an adjunct to DM, although these results were not statically significant. Full results from the trials (TOMMY^36^ and Victoria^29^) are not yet available; however, preliminary results suggest that use of DBT in assessment could lead to superior lesion localisation, significant reduction in unnecessary biopsies and could also reduce additional workup views^17^ and ultrasound^29^ requirements.

### Lesion Visibility

Inherent low X‐ray attenuation differences among cancerous and non‐cancerous glandular, stromal fibroglandular and epithelial tissues of the breast increase the complexity of the assessment in breast cancers. The characteristics of radiographic morphology of the breast that have been considered as risk indicators are:

1. Peripheral characteristics:
  1.1 Parenchymal pattern (formed mostly by functional/glandular tissues)
  1.2 Breast density
2. Focal characteristics:
  2.1 Masses non‐specific density (NSD)
  2.2 Architectural distortion (AD)
  2.3 Calcifications

Suspicious lesions, broadly classified into four categories – mass (with or without calcifications), calcifications, NSD and AD, manifest themselves in various patterns (such as spiculated masses, clustered calcifications) that radiologists recognise. Accurate identification, followed by dismissal if normal, or biopsy of suspicious lesions of the breast, contribute to the successful assessment of breast cancer.^40^, ^41^

DBT has been shown to provide superior lesion visibility due to reduced tissue superimposition (overlap effect).^4^, ^6^, ^7^, ^8^, ^10^, ^19^, ^21^ Studies have asserted a statistically significant improvement in visibility of breast tissues,^19^ particularly for spiculated lesions,^37^ whereas others determined a statistically significant improvement in visibility of malignant cases only.^8^ Reports of improved definition of multifocal,^2^ multicentric,^2^ or bilateral invasive lobular carcinoma (ILC) imply an efficient upfront assessment of these cases.^27^ Waldheer et al.^20^ have also argued that delineation of radial distortions of low‐density lesions, demarcation of small lesions and assessment of lesion margins is superior in DBT. Improved visibility of directionally oriented texture pattern converging towards the nipple,^42^ alongside reduction in structural noise and volumetric visualisation is the key to such superiority. Improved conspicuity in DBT has also been associated with enhanced background‐to‐lesion contrast resulting from reconstruction algorithms.^43^, ^44^

The effect of DBT on various radiographic morphology characteristics of lesions has been summarised in Table 1.

**Table 1:** The effect of digital breast tomosynthesis (DBT) on various radiographic morphology characteristics of lesions

	Advantages	Disadvantages	Conflicting findings
Masses	Improved visibility as compared to digital mammography (DM)^12^, ^27^, ^42^, ^48^, ^49^, ^50^ Can detect masses of size larger than 8 mm^37^, ^51^ Can detect spiculated mass^2^ of about 7 mm^2^ Spiculated masses are clearly seen^44^ Superior to DM in lesion size evaluation^35^	Loss of edge characteristics may be seen on low radiation dose DBT images	Does not lead to improved detection in invasive lobular carcinoma (ILC) 10; however another study has reported that ILC in DBT are clearly seen as spiculated masses^44^
Calcification		Loss in calcification characteristics and morphology may be seen^1^, ^10^, ^42^	Inferior performance of DBT as compared to DM^49^, ^50^ has been reported; although one study has also reported comparable performance^56^
Non‐specific density	Improved visibility as compared to DM^12^, ^27^, ^42^, ^49^, ^50^ Focal density is often presented as an ill‐defined mass^33^		
Architectural distortion (AD)	Improved visibility as compared to DM^27^, ^49^, ^52^ Reduced pseudo‐AD effects^42^ Improved detectability in DBT as compared to DM and other workup imaging techniques^52^		

#### Mass

According to the BI‐RADS standards,^45^ masses are defined as convex space‐occupying lesions seen in at least two orthogonal views. Masses are often benign. The shape, texture and appearance of the surrounding region, in conjunction with the mass itself, are used to identify suspicious lesions. According to Sickles, although multinodular or spiculated margins can sometimes be associated with malignancy, a poorly defined mass with irregular contours is a more common early sign of malignancy.^46^, ^47^ It was also observed that masses surrounded by radiodense fibroglandular tissue and containing no calcifications are often hard to detect on mammograms.^46^ The overlap effect explains the confounded and restricted visibility of lesions caused by the surrounding tissues, which may also obscure the irregular contours of lesions.

DBT, with the potential to eliminate the confounding information caused by structural noise, has been found to improve the visibility of masses.^12^, ^27^, ^42^, ^48^, ^49^, ^50^ Even at low contrast resolution, DBT can detect masses of size larger than 8 mm,^37^, ^51^ a size that was often missed by DM. Studies have shown that DBT could detect a spiculated mass^2^ of about 7 mm, for which the DM shows no suspicious finding.^2^ It has also been noted that spiculated masses that are often missed on DM are more clearly seen in DBT.^44^ Furthermore, in an assessment scenario, DBT is statistically significantly superior to DM in lesion size evaluation, which may be an important consideration for biopsy and surgical management.^35^

Loss of edge characteristics of lesions has been reported with very low‐radiation dose acquisition in DBT. This is mostly a problem in case of calcifications, however, low dose acquisition of malignant masses that become circumscribed over a period of time^42^ may result in borderline cases being misdiagnosed. It may also explain why DBT does not lead to improved detection in ILC.^10^ In DM, ILC often presents itself as a spiculated mass, however, it may also appear as an area of AD, NSD and, sometimes, it can even be radiographically occult.^44^ A side‐by‐side study of discrepant cases (cases that were marked by the same reader as suspicious in one modality but negative or probably benign in other modality) has found that ILC in DBT are clearly seen as spiculated masses. A recent study^27^ specifically evaluated ILC and reported that DBT significantly improves the detection ILC (both calcified and non‐calcified lesions). Edge determination and more precise lesion definition by DBT have the potential to greatly influence patient care as it may improve biopsy confidence and technique.

#### NSD

BI‐RADS^45^ defines asymmetry as planar, interspersedly fatty and lacking 3D conspicuity and convexity. Focal and global are the two types of asymmetric NSD seen in breasts. Global asymmetry is observed more commonly and is considered normal unless the degree of asymmetry is too high, additional mammographic signs are present and/or the patient is symptomatic. On the other hand, focal asymmetry is often concerning and is more problematic in dense breasts, where surrounding glandular tissue or benign masses may obscure a tumour. Accompanying distortion may cause retraction of normal tissue.^46^ It has been reported that the focal density in DBT is often presented as an ill‐defined mass.^33^ Studies^12^, ^27^, ^42^, ^49^, ^50^ have found that the 3D nature of DBT assists in improved visibility of NSD.

#### AD

Because of its inherent 3D nature, DBT not only improves the visibility of ADs^27^, ^49^, ^52^ but also reduces the pseudo‐AD effects.^42^ Reportedly, ADs were often missed in DM.^53^, ^54^ With the aid of DBT, ADs that would otherwise remain undetected in DM can be revealed.^52^ Improved detectability of AD in DBT as compared to other workup imaging techniques such as SVM, US and MRI (Table 2) increases the possibility of use of DBT in assessment. Improved performance of DBT in detecting AD over DM is a significant advance as AD is the leading characteristic of missed cancers.^42^

**Table 2:** Number of mammographically occult ADs revealed by various imaging techniques, as reported by Partyka et al.^52^

AD seen in	Total number of ADs	Spot‐view mammogram	Ultrasonography	Magnetic resonance imaging	Digital breast tomosynthesis (DBTs)
Both digital mammography (DM) and DBT	1	–	–	–	1
Better in DBT compared to DM alone	6	5	4	–	6
DBT alone	19	7	6	3	19

#### Calcifications

Sickles, in his ‘mammographic features of early breast cancer’ studies, has identified the importance of the presence of calcifications in breast disease.^46^ In spite of being mostly benign and potentially hard to detect, calcifications, based on shapes, are often a good indicator of malignancy.^55^ Sigfusson et al.^55^ have pointed out that round and oval‐shaped calcifications often indicate benign processes, whereas linear and branching shapes are an indicator for malignant lesions. DM^53^ is observed to be better at detecting calcification as compared to DBT.^49^, ^50^ This has also been associated with unnecessary recall, false negative results^4^ and inferior detectability of ductal carcinoma in situ (DCIS)^13^ in DBT.

Although calcifications (and their morphology^42^) are visible in DBT,^1^, ^10^ the out of plane blurring pare off the volumetric distribution of calcifications from individual slices, resulting into a loss in calcification characteristics and morphology. The detector^48^ and reconstruction noise in DBT are also known to affect micro‐calcifications visualisation. Conversely, based on another study involving 2–300 micron particle sizes, suspicious (pleomorphic, clustered or segmental) calcifications are equally (and in some cases superiorly) seen in DBT as compared to mammography.^56^ It has been argued that the other projection views of DBT could potentially improve the calcification visibility.^50^ Viewing multiple DBT slices together (slab technique) and then employing a maximum intensity projection post‐processing technique could potentially improve the visibility of micro‐calcification clusters.^56^ A reduction in pixel binning and reconstruction noise could potentially improve the detectability of calcifications in DBT.^42^

In spite of the poor visibility of calcifications, the diagnostic performance of DBT, as measured by AUC, did not differ significantly when compared to DM.^57^ In practice, DBT during assessment is still helpful in providing information on the distribution of calcifications, despite imperfect resolution. Calcifications may be identified as scattered through the breast on multiple slices, tightly grouped on a single slice or slab or in linear arrangement.

High case selection bias towards DM^57^ suggests lack of understanding of whether DBT can detect calcifications that were not revealed in DM. Future studies in DBT should evaluate the detectability of calcifications and ascertain whether use of super‐resolution techniques can improve their visibility.

## Limitations of DBT

Visibility of some lesions (masses and calcifications) may be adversely affected by the low‐radiation dose acquisition in DBT. This limitation, however, can be mitigated in the assessment clinic, as a small increase in the radiation dose is acceptable in favour of accurate lesion assessment. Detectability of calcifications still remains questionable in DBT^49^, ^50^ due to loss in calcification characteristics and morphology from out of plane blurring. Concerns that lesions may rarely be downgraded with use of DBT have also been raised,^37^ albeit for symptomatic breasts only.^58^

Radial scars, a benign sclerosing lesion, have superior visibility in DBT as compared to DM^59^ but may mimic a malignant mass‐like appearance.^60^ This may increase the number of false positives in DBT in assessment and thereby increase the number of unnecessary biopsies.

## Could DBT Assist in Biopsy?

Use of DBT in assisting biopsy is yet another area of exploration. During an assessment examination, tissue samples of possibly malignant lesions may be acquired with the help of fine‐needle aspiration (FNA) (ultrasound‐guided biopsy, stereotactic needle biopsy (SNB)) or core biopsy procedures (including vacuum assisted biopsy (VAB)). Lesion localisation is critical to this procedure as failure to accurately obtain the appropriate tissue sample either leads to multiple invasive procedures or incorrect or ineffective tissue sampling. DBT has the potential to detect lesions that are not visible on mammography. Therefore, DBT may have an important role to play in the sampling of such tissues. Furthermore, DBT has the potential to assist with not only accurate lesion localisation^61^ but also needle path planning^62^, ^63^ due to its 3D capabilities. Few studies have focused on evaluating DBT in a biopsy scenario.

One study with a sample size of 205 women reported a 100% success rate in tissue sampling with DBT‐guided VAB, with 43% reduction in procedural time,^61^, ^64^, ^65^ with fewer acquisitions and without causing any complications as compared to mammography‐guided VAB.^61^ Another recent study has seconded these results while also reporting superior performance in targeting ADs.^64^ DBT‐guided VAB was also reported to be successful in extracting clusters of fine calcifications^64^ that mammography‐guided VAB failed to achieve.^61^ These results suggest that the biopsy co‐ordinates obtained for DBT‐guided VAB were accurate and the technique to calculate biopsy co‐ordinates from the most suitable DBT slice was superior to traditional triangulation methods used in mammography‐guided VAB. Initial results are very promising and suggest that DBT has the potential to assist with superior lesion localisation^61^ and can be effective in biopsy as well, however, further research is required to understand the efficacy of DBT in biopsy.

An optimal needle path planning is the key to successful and convenient tissue sampling. An inaccurate needle path planning often leads to complications such as haematoma or the need for multiple attempts at tissue sampling. In 100% of the cases, DBT‐guided VAB successfully obtained tissue samples in the first attempt.^61^ Hologic, Inc. has proposed the use of DBT in biopsy and needle localisation capitalising on the 3D localisation capabilities and high‐contrast visibility of DBT.^63^ Another study has proposed a probabilistic model for needle localisation using DBT.^62^ Clip deployment as a marker of the biopsy site can be performed at the end of the DBT biopsy procedure, with clip position documented on two‐view standard mammography. However, to our knowledge, only one study^61^ has reported use of DBT in VAB and other areas of biopsy such as FNA, SNB are still largely unstudied.

## Conclusion

Although the results from population‐based trials are forthcoming, initial results on use of DBT in assessment have been positive. The superior performance of DBT in demarcation of mass, density and ADs could potentially reduce the complexity of assessment by confirming normality and reducing the number of required workup views, by increasing the diagnostic accuracy of mammographic lesion evaluation and by increasing the radiologists’ confidence on diagnosis. The 3D capability of DBT enables its use in biopsy and related procedures (e.g. optimal needle path planning). Early results indicate that DBT has the potential for successful adaptation in biopsy.

## Conflict of Interest

The authors declare no conflict of interest.

## References

[1] Kopans DB . Breast Imaging, 3rd edn. Lippincott Williams & Wilkins, Philadelphia, PA, 2007.

[2] Hologic Inc . The use of breast tomosynthesis in clinical practice. Applied Radiology, 2012 Available from: http://www.hologic.com/sites/default/files/white-papers/2012 Nov ‐ Applied Radiology Tomosynthesis Supplement ‐ FINAL.pdf.)

[3] Alakhras M , Bourne R , Rickard M , Ng KH , Pietrzyk M , Brennan PC . Digital tomosynthesis: A new future for breast imaging? Clin Radiol 2013; 68: e225–36. 23465326 10.1016/j.crad.2013.01.007

[4] Poplack SP , Tosteson TD , Kogel CA , Nagy HM . Digital breast tomosynthesis: Initial experience in 98 women with abnormal digital screening mammography. Am J Roentgenol 2007; 189: 616–23. 17715109 10.2214/AJR.07.2231

[5] Michell MJ , Iqbal A , Wasan RK , et al. A comparison of the accuracy of film‐screen mammography, full‐field digital mammography, and digital breast tomosynthesis. Clin Radiol 2012; 67: 976–81. 22625656 10.1016/j.crad.2012.03.009

[6] Good WF , Abrams GS , Catullo VJ , et al. Digital breast tomosynthesis: A pilot observer study. Am J Roentgenol 2008; 190: 865–9. 18356430 10.2214/AJR.07.2841

[7] Wallis MG , Moa E , Zanca F , Leifland K , Danielsson M . Two‐view and single‐view tomosynthesis versus full‐field digital mammography: High‐resolution X‐ray imaging observer study. Radiology 2012; 262: 788–96. 22274840 10.1148/radiol.11103514

[8] Andersson I , Ikeda DM , Zackrisson S , et al. Breast tomosynthesis and digital mammography: A comparison of breast cancer visibility and BIRADS classification in a population of cancers with subtle mammographic findings. Eur Radiol 2008; 18: 2817–25. 18641998 10.1007/s00330-008-1076-9

[9] Svane G , Azavedo E , Lindman K , et al. Clinical experience of photon counting breast tomosynthesis: comparison with traditional mammography. Acta Radiol 2011; 52: 134–42. 21498340 10.1258/ar.2010.100262

[10] Teertstra HJ , Loo CE , van den Bosch MAAJ , et al. Breast tomosynthesis in clinical practice: Initial results. Eur Radiol 2010; 20: 16–24. 19657655 10.1007/s00330-009-1523-2

[11] Gennaro G , Toledano A , di Maggio C , et al. Digital breast tomosynthesis versus digital mammography: A clinical performance study. Eur Radiol 2010; 20: 1545–53. 20033175 10.1007/s00330-009-1699-5

[12] Hakim CM , Chough DM , Ganott MA , Sumkin JH , Zuley ML , Gur D . Digital breast tomosynthesis in the diagnostic environment: A subjective side‐by‐side review. Am J Roentgenol 2010; 195: W172–6. 20651178 10.2214/AJR.09.3244

[13] Svahn TM , Chakraborty DP , Ikeda D , et al. Breast tomosynthesis and digital mammography: A comparison of diagnostic accuracy. Br J Radiol 2012; 85: E1074–82. 22674710 10.1259/bjr/53282892 PMC3500806

[14] Rafferty EA , Park JM , Philpotts LE , et al. Assessing radiologist performance using combined digital mammography and breast tomosynthesis compared with digital mammography alone: Results of a multicenter, multireader trial. Radiology 2013; 266: 104–13. 23169790 10.1148/radiol.12120674 PMC5410947

[15] Ciatto S , Houssami N , Bernardi D , et al. Integration of 3D digital mammography with tomosynthesis for population breast‐cancer screening (STORM): A prospective comparison study. Lancet Oncol 2013; 14: 583–9. 23623721 10.1016/S1470-2045(13)70134-7

[16] Skaane P , Bandos AI , Gullien R , et al. Prospective trial comparing full‐field digital mammography (FFDM) versus combined FFDM and tomosynthesis in a population‐based screening programme using independent double reading with arbitration. Eur Radiol 2013; 23: 2061–71. 23553585 10.1007/s00330-013-2820-3 PMC3701792

[17] Noroozian M , Hadjiiski L , Rahnama‐Moghadam S , et al. Digital breast tomosynthesis is comparable to mammographic spot views for mass characterization. Radiology 2012; 262: 61–8. 21998048 10.1148/radiol.11101763 PMC3244671

[18] Gennaro G , Hendrick RE , Ruppel P , et al. Performance comparison of single‐view digital breast tomosynthesis plus single‐view digital mammography with two‐view digital mammography. Eur Radiol 2013; 23: 664–72. 22976919 10.1007/s00330-012-2649-1

[19] Gennaro G , Hendrick RE , Toledano A , et al. Combination of one‐view digital breast tomosynthesis with one‐view digital mammography versus standard two‐view digital mammography: Per lesion analysis. Eur Radiol 2013; 23: 2087–94. 23620367 10.1007/s00330-013-2831-0

[20] Waldherr C , Cerny P , Altermatt HJ , et al. Value of one‐view breast tomosynthesis versus two‐view mammography in diagnostic workup of women with clinical signs and symptoms and in women recalled from screening. Am J Roentgenol 2013; 200: 226–31. 23255766 10.2214/AJR.11.8202

[21] Gur D , Abrams GS , Chough DM , et al. Digital breast tomosynthesis: Observer performance study. Am J Roentgenol 2009; 193: 586–91. 19620460 10.2214/AJR.08.2031

[22] Smith AP , Rafferty EA , Niklason L . Clinical performance of breast tomosynthesis as a function of radiologist experience level In: Krupinski EA (ed). Digital Mammography. IWDM 2008. Lecture Notes in Computer Science, Vol 5116. Springer, Berlin, 2008.

[23] Gur D , Bandos AI , Rockette HE , et al. Localized detection and classification of abnormalities on FFDM and tomosynthesis examinations rated under an FROC paradigm. Am J Roentgenol 2011; 196: 737–41. 21343521 10.2214/AJR.10.4760

[24] Tingberg A , Fornvik D , Mattsson S , Svahn T , Timberg P , Zackrisson S . Breast cancer screening with tomosynthesis‐initial experiences. Radiat Prot Dosimetry 2011; 147: 180–3. 21733859 10.1093/rpd/ncr296

[25] Gilbert FJ , Gillan MGC , Michell MJ , et al. TOMMY Trial (a comparison of tomosynthesis with digital mammography in the UK NHS breast screening programme) setting up a multicentre imaging trial. Breast Cancer Res 2011; 13. 10.3310/hta19040 PMC4781321 25599513

[26] Skaane P , Bandos AI , Gullien R , et al. Comparison of digital mammography alone and digital mammography plus tomosynthesis in a population‐based screening program. Radiology 2013; 267: 47–56. 23297332 10.1148/radiol.12121373

[27] Mariscotti G , Durando M , Houssami N , et al. Digital breast tomosynthesis as an adjunct to digital mammography for detecting and characterising invasive lobular cancers: A multi‐reader study. Clin Radiol 2016; 71: 889–95. 27210245 10.1016/j.crad.2016.04.004

[28] Fornvik D , Andersson I , Svahn T , Timberg P , Zackrisson S , Tingberg A . The effect of reduced breast compression in breast tomosynthesis: Human observer study using clinical cases. Radiat Prot Dosimetry 2010; 139: 118–23. 20228049 10.1093/rpd/ncq103

[29] Lockie D , Nickson C , Aitken Z . Evaluation of digital breast tomosynthesis (DBT) in an Australian BreastScreen assessment service (an abstract). J Med Radiat Sci 2014; 61: 63–112.

[30] Alakhras M , Mello‐Thoms C , Rickard M , Bourne R , Brennan PC . Efficacy of digital breast tomosynthesis for breast cancer diagnosis. Proc SPIE 9037, Medical Imaging 2014: Image Perception, Observer Performance, and Technology Assessment, 90370V (March 11, 2014; 2014. p. 90370V‐V‐9.

[31] Reed WM , Ryan JT , McEntee MF , Evanoff MG , Brennan PC . The effect of abnormality‐prevalence expectation on expert observer performance and visual search. Radiology 2011; 258: 938–43. 21248231 10.1148/radiol.10101090

[32] Evans KK , Birdwell RL , Wolfe JM . If you don't find it often, you often don't find it: Why some cancers are missed in breast cancer screening. PLoS ONE 2013; 8: e64366. 23737980 10.1371/journal.pone.0064366 PMC3667799

[33] Yang T‐L , Liang H‐L , Chou C‐P , Huang J‐S , Pan H‐B . The adjunctive digital breast tomosynthesis in diagnosis of breast cancer. Biomed Res Int 2013; 2013: 597253. 23844366 10.1155/2013/597253 PMC3703369

[34] Tagliafico A , Astengo D , Cavagnetto F , et al. One‐to‐one comparison between digital spot compression view and digital breast tomosynthesis. Eur Radiol 2012; 22: 539–44. 21987214 10.1007/s00330-011-2305-1

[35] Mun HS , Kim HH , Shin HJ , et al. Assessment of extent of breast cancer: Comparison between digital breast tomosynthesis and full‐field digital mammography. Clin Radiol 2013; 68: 1254–9. 23969151 10.1016/j.crad.2013.07.006

[36] Gilbert FJ , Duffy SW , Gillan MGC , et al. TOMMY Trial (a comparison of tomosynthesis with digital mammography in the UK NHS breast screening programme) setting up a multicentre imaging trial. Breast Cancer Res 2011;13 Suppl 1: P28‐P.

[37] Clark G , Valencia A . Does tomosynthesis increase confidence in grading the suspicious appearance of a lesion? An audit of cancers diagnosed in the assessment clinic using tomosynthesis: Initial experience at Avon Breast Screening Unit. Breast Cancer Res 2015; 17: 2.25572591

[38] Chae EY , Kim HH , Cha JH , Shin HJ , Choi WJ . Detection and characterization of breast lesions in a selective diagnostic population: Diagnostic accuracy study for comparison between one‐view digital breast tomosynthesis and two‐view full‐field digital mammography. Br J Radiol 2016; 89: 8. 10.1259/bjr.20150743 PMC5258147 27072391

[39] Svahn T , Andersson I , Chakraborty D , et al. The diagnostic accuracy of dual‐view digital mammography, single‐view breast tomosynthesis and a dual‐view combination of breast tomosynthesis and digital mammography in a free‐response observer performance study. Radiat Prot Dosimetry 2010; 139: 113–7. 20228048 10.1093/rpd/ncq044 PMC2911156

[40] Wolfe JN . Breast patterns as an index of risk for developing breast‐cancer. Am J Roentgenol 1976; 126: 1130–9. 179369 10.2214/ajr.126.6.1130

[41] Sickles EA . Mammographic features of 300 consecutive nonpalpable breast cancers. Am J Roentgenol 1986; 146: 661–3. 3485337 10.2214/ajr.146.4.661

[42] Uematsu T . The emerging role of breast tomosynthesis. Breast Cancer 2013; 20: 204–12. 23456738 10.1007/s12282-013-0456-4

[43] Tingberg A . X‐ray tomosynthesis: A review of its use for breast and chest imaging. Radiat Prot Dosimetry 2010; 139: 100–7. 20233756 10.1093/rpd/ncq099

[44] Lång K , Andersson I , Zackrisson S . Breast cancer detection in digital breast tomosynthesis and digital mammography—a side‐by‐side review of discrepant cases. Br J Radiol 2014; 87: 20140080. 24896197 10.1259/bjr.20140080 PMC4112403

[45] Sickles E , D'Orsi CJ , Bassett LW , et al. ACR BI‐RADS^®^ Mammography In: ACR BI‐RADS^®^ Atlas, Breast Imaging Reporting and Data System. American College of Radiology, Reston, VA, 2013.

[46] Sickles EA . Mammographic features of early breast‐cancer. Am J Roentgenol 1984; 143: 461–4. 6331721 10.2214/ajr.143.3.461

[47] Moskowitz M . Minimal breast‐cancer redux. Radiol Clin North Am 1983; 21: 93–113. 6836105

[48] Nishikawa RM , Reiser I , Seifi P . A new approach to digital breast tomosynthesis for breast cancer screening. Medical Imaging 2007 Conference, 18–20 February 2007, San Diego, CA, pp. U1464–U71.

[49] Brandt KR , Craig DA , Hoskins TL , et al. Can digital breast tomosynthesis replace conventional diagnostic mammography views for screening recalls without calcifications? A comparison study in a simulated clinical setting. Am J Roentgenol 2013; 200: 291–8. 23345348 10.2214/AJR.12.8881

[50] Rafferty EA . Digital mammography: Novel applications. Radiol Clin North Am 2007; 45: 831–43. 17888772 10.1016/j.rcl.2007.06.005

[51] Timberg P , Bath M , Andersson I , Mattsson S , Tingberg A , Ruschin M . Visibility of microcalcification clusters and masses in breast tomosynthesis image volumes and digital mammography: A 4AFC human observer study. Med Phys 2012; 39: 2431–7. 22559613 10.1118/1.3694105

[52] Partyka L , Lourenco AP , Mainiero MB . Detection of mammographically occult architectural distortion on digital breast tomosynthesis screening: Initial clinical experience. Am J Roentgenol 2014; 203: 216–22. 24951218 10.2214/AJR.13.11047

[53] Lewin JM , Hendrick RE , D'Orsi CJ , et al. Comparison of full‐field digital mammography with screen‐film mammography for cancer detection: Results of 4945 paired examinations. Radiology 2001; 218: 873–80. 11230669 10.1148/radiology.218.3.r01mr29873

[54] Lewin JM , D'Orsi CJ , Hendrick RE , et al. Clinical comparison of full‐field digital mammography and screen‐film mammography of breast cancer. Am J Roentgenol 2002; 179. 10.2214/ajr.179.3.1790671 12185042

[55] Sigfusson BF , Andersson I , Aspegren K , Janzon L , Linell F , Ljungberg O . Clustered breast calcifications. Acta Radiol Diagn 1983; 24: 273–81. 10.1177/028418518302400401 6637566

[56] Kopans D , Gavenonis S , Halpern E , Moore R . Calcifications in the breast and digital breast tomosynthesis. Breast J 2011; 17: 638–44. 21906207 10.1111/j.1524-4741.2011.01152.x

[57] Spangler ML , Zuley ML , Sumkin JH , et al. Detection and classification of calcifications on digital breast tomosynthesis and 2D digital mammography: A comparison. Am J Roentgenol 2011; 196: 320–4. 21257882 10.2214/AJR.10.4656

[58] Bansal GJ , Young P . Digital breast tomosynthesis within a symptomatic “one‐stop breast clinic” for characterization of subtle findings. Br J Radiol 2015; 88: 20140855. 26133221 10.1259/bjr.20140855 PMC4743564

[59] Skaane P , Gullien R , Eben E , et al. Radial scar: a diagnostic challenge in breast cancer screening using tomosynthesis. Radiological Society of North America 2013 Scientific Assembly and Annual Meeting; 2013; Chicago IL. Available from: http://archive.rsna.org/2013/13016491.html Accessed May 12, 2016.

[60] Freer PE , Wang JL , Rafferty EA . Digital breast tomosynthesis in the analysis of fat‐containing lesions. Radiographics 2014; 34: 343–58. 24617683 10.1148/rg.342135082

[61] Schrading S , Distelmaier M , Dirrichs T , et al. Digital breast tomosynthesis–guided vacuum‐assisted breast biopsy: Initial experiences and comparison with prone stereotactic vacuum‐assisted biopsy. Radiology 2015; 274: 654–62. 25386875 10.1148/radiol.14141397

[62] Vancamberg L , Sahbani A , Muller S , Morel G . Needle path planning method for digital breast tomosynthesis biopsy based on probabilistic techniques In: Marti J, Oliver A, Freixenet J, Marti R (eds). Digital Mammography. *IWDM 2010* Lecture Notes in Computer Science, vol 6136. Springer, Berlin, 2010; 15–22.

[63] DeFreitas KF , Shaw I , Laviola J , et al. Breast biopsy and needle localization using tomosynthesis systems. Official Gazette of the United States Patent and Trademark Office Patents 2013.

[64] Waldherr C , Berclaz G , Altermatt HJ , et al. Tomosynthesis‐guided vacuum‐assisted breast biopsy: A feasibility study. Eur Radiol 2016; 26: 1582–9. 26385802 10.1007/s00330-015-4009-4

[65] Munir A , Huws A , Moalla A , et al. Our initial experience of digital breast tomosynthesis guided vacuum assisted breast biopsies and the patient's perspective: A single centre experience. Eur J Surg Oncol 2016; 42: S11.

